# Gender Differences in the Clinical Profile of Sodium-Glucose Cotransporter-2 Inhibitor-Related Urinary Tract Infections

**DOI:** 10.7759/cureus.67590

**Published:** 2024-08-23

**Authors:** Mohammad Hayat Bhat, Mohammad Salem Baba, Md Ejaz Alam, Abid Hussain Bhat, Shahnawaz Mir, Basharat Qayoom Dar, Shoiab Mohd Patto, Pooran Sharma

**Affiliations:** 1 Department of Endocrinology, Government Superspeciality Hospital, Srinagar, IND; 2 Department of Endocrinology, Diabetes and Metabolism, Government Medical College, Srinagar, Srinagar, IND

**Keywords:** suti, clinical profile, gender, uti, sglt2i

## Abstract

Background

Sodium-glucose cotransporter-2 inhibitors (SGLT2Is) are a novel class of oral antidiabetic agents with proven cardiovascular and mortality benefits. By promoting glucosuria, SGLT2Is increase the risk of genital and urinary tract infections (UTIs), which remain uncomplicated in most cases. Comparative studies detailing the gender differences in the clinical profile of SGLT2I-related UTIs (SUTIs) are lacking. Hence, this study was designed to investigate the gender-related differences in the clinical profile of SUTIs.

Methodology

This prospective study enrolled 100 consecutive diabetes mellitus patients with UTI symptoms who were on SGLT2Is. In addition to collecting clinical details, patients were subjected to the following investigations: complete blood counts, urea, creatine, liver function, lipid components, urine analysis, urine culture, and ultrasonography.

Results

Females (n = 80) outnumbered males (n = 20). Although females were younger than males (53.68 ± 10.26 vs. 63.30 ± 10.75 years, p = 0.003), body mass index (29.84 ± 7.22 vs. 24.62 ± 3.10 kg/m^2^, p = 0.008) and waist circumference (103.01 ± 14.49 vs. 93.75 ± 4.50 cm, p = 0.109) were higher in females. About 22.5% of females had undergone hysterectomy. The mean duration of diabetes mellitus was longer in males (10.64 ± 6.74 vs. 7.78 ± 4.75 years), whereas the median duration of SGLT2I use (4 (interquartile range (IQR) = 1-12) vs. 3 (IQR = 2-4) months) and mean HbA1c levels were not different between the two groups. A greater proportion of males were complicated by retinopathy (55% vs. 15%) and proteinuria (65% vs. 17.5%), while neuropathy (85% vs. 71.25%) rates were similar. Overall, 35% of males had complicated UTIs (renal abscess, pyelonephritis, prostatic abscess) compared to only 3.75% of females (p = 0.001).

Conclusions

The majority of SUTIs are uncomplicated in females whereas in males one-third are complicated infections. Although females with SUTI had a higher prevalence of obesity and dyslipidemia, males had a longer duration of diabetes mellitus and higher retinopathy prevalence. Extreme caution should be exercised in patients at risk for SUTI before prescribing SGLT2I.

## Introduction

Sodium-glucose cotransporter-2 inhibitors (SGLT2Is) are a new class of oral antidiabetic agents with proven cardiovascular and mortality benefits [1]. They promote glucosuria by predominantly acting on renal tubules, thereby increasing the risk of genital and urinary tract infections (UTIs). This increased risk of UTI is consistent across all SGLT2Is such as dapagliflozin, empagliflozin, and canagliflozin [2-4]. Nevertheless, the majority of these episodes are mild and do not warrant therapy discontinuation. The risk factors for SGLT2I-related UTI (SUTI) are older age, female sex, poor glycemic control, dyslipidemia, and the presence of microvascular complications [5-9]. Some case reports suggest that SUTI may have adverse consequences, especially in males [10]. However, comparative studies detailing the gender differences in the clinical profile of SUTIs are lacking. Therefore, this study was designed to characterize the gender-related differences in the clinical profile of SUTIs.

## Materials and methods

This prospective study recruited all consecutive patients with diabetes mellitus (DM) attending the outpatient department of a tertiary care hospital (Government Medical College, Srinagar) between March 2023 and September 2023 with symptoms of UTI and were receiving SGLT2Is. Written informed consent was obtained from each study participant, and the study was approved by the Institutional Ethics Committee of Government Medical College, Srinagar (approval number: IRBGMC-SGR/Endo/512).

Clinical assessment

The participants were subjected to a detailed clinical assessment. Relevant medical histories were obtained, including duration of DM, duration of SGLT2I use, previous UTI episodes, and the presence of comorbidities such as hypertension (HTN), dyslipidemia, non-alcoholic fatty liver disease (NAFLD), obesity, obstructive sleep apnea (OSA), a history of hysterectomy in females, and a review of previous records for complication assessment. The complication status of DM, such as neuropathy, proteinuria, and retinopathy, was based on previous assessments conducted within one year. The current history was noted for symptoms of UTI or its complications (dysuria, increased frequency of micturition, fever, pain in the abdomen, and altered sensorium). Physical examination included measurements of blood pressure (BP), height, weight, waist circumference (WC), and hip circumference (HC). The anthropometric measurements were performed by two examiners (MSB, AA) using standard instruments and light clothing. A comprehensive systemic examination was also performed.

Inclusion and exclusion criteria

We included patients diagnosed with DM who were currently receiving SGLT2I therapy and presented with symptoms of UTI. We excluded pregnant women, patients on indwelling urinary catheters, patients with a recent history of antibiotic treatment (within two weeks), patients on prophylaxis for recurrent UTIs, and immunocompromised patients (HIV, malignancy, patients on steroids, and transplant recipients).

Laboratory measurements

The participants were asked to provide a random blood sample for the following investigations: hemoglobulin (Hb), total leukocyte count (TLC), platelet count, urea, creatinine, bilirubin, alanine aminotransferase (ALT), alkaline phosphatase (ALP), total protein, albumin, glucose, glycated hemoglobin (HbA1c), total cholesterol (TC), high-density lipoprotein (HDL) cholesterol, low-density lipoprotein (LDL) cholesterol, triglycerides (TG), calcium (Ca), phosphorous (PO_4_), and uric acid (UA). The blood was allowed to clot at room temperature (15-25°C) and centrifuged for 15 minutes to obtain hemolysis-free serum.

Urea, creatinine, bilirubin, ALT, ALP, total protein, albumin, glucose, HbA1c, lipid profile, Ca, PO_4_, and uric acid were estimated on the same day using an automated chemistry analyzer (Hitachi-912, Tokyo, Japan). Lipid parameters were analyzed the same day with commercially available enzymatic reagents (Audit Diagnostics, Ireland) adapted to the Hitachi-912 autoanalyzer. HbA1c was measured using high-performance liquid chromatography standardized to the Diabetes Control and Complications Trial (DCCT) assay on an Avantor A9 HbA1c analyzer with whole blood collected in an ethylenediaminetetraacetic acid tube. After proper education, a voided midstream urine sample was collected in a clean and sterile container for routine urine analysis and culture sensitivity. Samples were inoculated on Hichrome UTI agar media to determine the colony-forming unit. The organisms were identified using standard culture techniques and morphological and biochemical parameters.

Definitions

The diagnosis of UTI was based on symptomology and abnormal urinalysis (>10 leukocytes per high-power field), regardless of culture reports. Being overweight was defined as a body mass index (BMI) of 23-27.4 kg/m^2^ and obesity as a BMI of 27.5 kg/m^2^.

Imaging

All patients were subjected to ultrasonography. Postvoidal residual urine (PURU) was measured after emptying the bladder. A total of 12 patients also underwent non-contrast CT of the abdomen in whom there was suspicion of pyelonephritis or abscess.

Statistical analysis

Statistical analysis was performed using SPSS Statistics version 20 (IBM Corp., Armonk, NY, USA). The normality of data was checked by the Kolmogorov-Smirnov test. Quantitative data were described as the mean and standard deviation for normally distributed data, while median and interquartile range (IQR) were used for non-normally distributed data. Categorical variables were described using frequency and percentage. To compare categorical variables, the chi-square test was employed. Student’s independent t-test and Mann-Whitney test were used for normally and non-normally distributed continuous variables, respectively. All results were considered significant at a 5% level of significance (p < 0.05).

## Results

A total of 100 patients with DM were recruited in this study, including 80 females and 20 males. The mean age of females (53.68 ± 10.46 years) was significantly lower than males (63.30 ± 10.75 years, p = 0.003), whereas mean BMI (29.84 ± 7.22 vs. 24.62 ± 3.10 kg/m^2^, p = 0.042 ) and mean WC (103.01 ± 14.49 vs. 93.75 ± 4.50 cm, p = 0.019) was higher in females than males. Although the mean duration of DM was longer in males (10.64 ± 6.74 vs. 7.78 ± 4.75 years, p = 0.042), the median duration of SGLT2I (4 (IQR = 1-12) vs. 3 (IQR = 2-4) months, p = 0.236) use was not different in the two groups. The mean Hba1c, creatinine, ALT, Hb, TLC, and platelet count were not different between the two groups. However, TC and TG were higher among females. On USG, median PURU was higher in males (65 (IQR = 35-82 mL) vs. 12 (IQR = 11-32 mL), p = 0.045) (Table 1).

**Table 1:** Comparison of anthropometric, clinical, and biochemical parameters of males and females. The data are presented as mean ± SD unless specified. ^#^: Median and interquartile range. SGLT2I: sodium-glucose cotransporter-2 inhibitor

Parameter	Males (n = 20)	Females (n = 80)	P-value
Age (years)	63.30 ± 10.75	53.68 ± 10.26	0.003
Weight (kg)	65.61 ± 7.75	78.49 ± 13.75	0.002
Body mass index (kg/m^2^)	24.62 ± 3.10	29.84 ± 7.22	0.008
Waist circumference (cm)	93.75 ± 4.50	103.01 ± 14.49	0.019
Duration of diabetes (years)	10.64 ± 6.74	7.78 ± 4.75	0.042
Duration of SGLT2I use (months)^#^	4 (1–12)	3 (2–4)	0.236
HbA1c (%)	9.58 ± 3.05	10.27 ± 12.89	0.868
Creatinine (mg/dL)	1.32 ± 0.24	1.14 ± 0.79	0.796
Albumin (mg/dL)	3.80 ± 0.39	3.85 ± 0.44	0.801
Serum alanine aminotransferase (IU/L)	22.85 ± 10.96	40.50 ± 33.52	0.185
Serum total cholesterol (mg/dL)	105.01 ± 11.74	152.10 ± 45.54	0.014
Serum triglycerides (mg/dL)	86.50 ± 35.80	183.43 ± 92.10	0.015
Serum low-density cholesterol (mg/dL)	61.50 ± 35.23	88.35 ± 31.25	0.146
Serum high-density cholesterol (mg/dL)	40.50 ± 9.74	39.22 ± 8.05	0.784
Hemoglobin (g/dL)	11.47 ± 1.66	10.9 ± 1.35	0.486
Total leucocyte count (×10^3^/L)	10.85 ± 3.88	7.87 ± 2.51	0.055
Platelets (×10^5^/L)	107.50 ± 74.99	180.72 ± 114.36	0.233
Postvoid residual urine (mL)^#^	65 (35–82)	12 (11–32)	0.045

Around 70% (n = 14) of males had HTN compared to 68.75% (n = 55) of females. Overall, 55% (n = 11) of males were overweight compared to 23.75% (n = 19) of females, while 67.5% (n = 54) of females were obese compared to 10% (n = 2) of males. NAFLD prevalence was higher in females (68.75% vs. 55%). Further, 6.25% (n = 5) of females had osteoporosis compared to one male (Table 2). A significantly higher number of males had retinopathy compared to females (55% (n = 11) vs. 15% (n = 12), p = 0.002). Similarly, proteinuria prevalence was also higher in males (65% (n = 13) vs. 17.5% (n = 14), p = 0.001). However, neuropathy (85% vs. 71.25%, p = 0.682) and cystopathy (50% vs. 36.25%, p = 0.963) were not different among males and females. About 23.4% of females had undergone hysterectomy in the past. Moreover, 26.25% of females had suffered a UTI in the past three months compared to 25% of males. Further, 35% of males had complicated UTI (renal abscess, pyelonephritis, prostatic abscess) compared to only 3.75% of females. Most patients were on dapagliflozin (n = 98), two were on empagliflozin, and none were on canagliflozin. Among females, cultures were available for 62 subjects (sterile in 49, *Escherichia coli* in 10, *Enterococcus* in two, coagulase-negative *Staphylococcus* in one) while cultures were available in 14 males (sterile in eight, *Escherichia coli* in four, *Klebsiella* in one, and *Enterococcus* in one).

**Table 2:** Comparison of comorbidities and complications between males and females. NAFLD: non-alcoholic fatty liver disease; OSA: obstructive sleep apnea; UTI: urinary tract infection; SGLT2I: sodium-glucose cotransporter-2 inhibitor

Parameter	Males (n = 20)	Females (n = 80)	P-value
Hypertension	14 (70.0%)	55 (68.75%)	0.555
Overweight	11 (55.0%)	19 (23.75%	0.011
Obesity	2 (10.0%)	54 (67.5%)	0.001
NAFLD	11 (55.0%)	55 (68.75%)	0.133
Retinopathy	11 (55.0%)	12 (15.0%)	0.002
Neuropathy	17 (85.0%)	57 (71.25%)	0.682
Proteinuria	13 (65.0%)	14 (17.5%)	0.001
Cystopathy	10 (50.0%)	29 (36.25%)	0.963
OSA	3 (15.0%)	29 (36.25%)	0.945
Osteoporosis	1 (5.0%)	5 (6.25%)	0.867
UTI in 3 months	5 (25.0%)	21 (26.25%)	0.339
Complicated UTI	7 (35.0%)	3 (3.75%)	0.001

Patients with complicated infections were older and had a longer duration of DM, while BMI, WC, duration of SGLT2I use, and HbA1c were not different from those with uncomplicated infections. However, patients with complicated infections had increased creatinine, TLC, and PURU (Table 3). On further subgroup analysis of females who had undergone hysterectomy, mean weight and BMI were lower in subjects who had undergone hysterectomy. At the same time, WC, HbA1c, duration of DM, duration of SGLT2I use, HbA1c, and creatinine were not different from those who had not undergone hysterectomy (Table 4).

**Table 3:** Comparison of anthropometric, clinical, and biochemical parameters depending on the type of UTI. The data are presented as mean ± SD unless specified. #: Median and interquartile range. SGLT2I: sodium-glucose cotransporter-2 inhibitor; UTI: urinary tract infection

parameter	Uncomplicated (n = 90)	Complicated (n = 10)	P-value
Age (years)	54.57 ± 10.56	62.50 ± 11.06	0.049
Weight (kg)	76.06 ± 12.13	72.75 ± 22.26	0.528
Body mass index (kg/m^2^)	27.68 ± 4.07	27.07 ± 10.06	0.754
Waist circumference (cm)	102.22 ± 12.32	89.33 ± 4.61	0.116
Duration of diabetes (years)	7.80 ± 4.67	11.81 ± 5.63	0.028
Duration of SGLT2I use (months)^#^	6 (2.65–6)	3 (0.75–3.5)	0.274
HbA1c (%)	10.13 ± 1.57	10.25 ± 3.13	0.979
Serum creatinine (mg/dL)	1.06 ± 0.43	1.82 ± 0.69	0.007
Serum albumin (mg/dL)	3.88 ± 0.49	3.85 ± 0.44	0.753
Serum alanine aminotransferase (IU/L)	37.90 ± 13.06	27.33 ± 10.52	0.400
Serum total cholesterol (mg/dL)	146.13 ± 42.94	132.01 ± 43.54	0.527
Serum triglycerides (mg/dL)	165.44 ± 52.80	171.40 ± 52.14	0.897
Serum low-density cholesterol (mg/dL)	88.23 ± 26.23	54.28 ± 23.35	0.021
Serum high-density cholesterol (mg/dL)	39.13 ± 7.54	41.43 ± 9.08	0.627
Hemoglobin (g/dL)	11.05 ± 2.66	11.09 ± 1.45	0.948
Total leukocyte count (×10^3^/L)	8.56 ± 3.38	10.48 ± 4.81	0.271
Platelets (×10^5^/L)	158.65 ± 71.59	185.66 ± 51.67	0.611
Post void residual urine (mL)^#^	12.5 (10–34)	50 (30–60)	0.018

**Table 4:** Comparison of anthropometric, clinical, and biochemical parameters of female subjects based on hysterectomy status. The data are presented as mean ± SD unless specified. #: Median and interquartile range. SGLT2I: sodium-glucose cotransporter-2 inhibitor

Parameter	Hysterectomy (n = 18)	No hysterectomy (n = 62)	P-value
Age (years)	54.13 ± 9.75	53.56 ± 10.56	0.853
Weight (kg)	70.46 ± 12.77	80.59 ± 13.350	0.030
Body mass index (kg/m^2^)	25.52 ± 4.63	29.54 ± 5.08	0.019
Waist circumference (cm)	94.50 ± 6.30	106.41 ± 15.40	0.358
Duration of diabetes (years)	9.46 ± 4.14	7.19 ± 4.65	0.093
Duration of SGLT2I use (months)^ #^	6 (2.5–6)	4 (2–6)	0.958
HbA1c	8.98 ± 1.53	10.72 ± 2.89	0.664
Serum creatinine (mg/dL)	1.10 ± 0.23	1.12 ± 0.79	0.940
Serum albumin (mg/dL)	3.79 ± 0.29	3.95 ± 0.54	0.395
Serum alanine aminotransferase (IU/L)	35.45 ± 12.96	40.75 ± 13.12	0.729
Serum total cholesterol (mg/dL)	148.51 ± 49.74	153.34 ± 40.54	0.822
Serum triglycerides (mg/dL)	165.23 ± 58.80	187.47 ± 42.10	0.631
Serum low-density cholesterol (mg/dL)	104.75 ± 34.23	83.25 ± 26.35	0.254
Serum high-density cholesterol (mg/dL)	39.80 ± 7.34	39.43 ± 8.08	0.876
Hemoglobin (g/dL)	11.38 ± 2.36	10.9 ± 1.45	0.654
Total leukocyte count (×10^3^/L)	7.89 ± 2.78	8.57 ± 3.81	0.456
Platelets (×10^5^/L)	187.25 ± 70.99	170.50 ± 78.36	0.685
Post void residual urine (mL)^#^	30 (13–42)	12 (10–12)	0.050

## Discussion

SUTI is one of the common complications of SGLT2Is, though the majority of these episodes are uncomplicated and do not warrant discontinuation of therapy. In this study, 100 patients with SUTI were studied, with three-fourths being females. While females with SUTI had higher BMI, WC, and a higher prevalence of NAFLD, males had a higher prevalence of retinopathy, proteinuria, and neuropathy.

Previous large population-based studies showed that female sex is a risk factor for SUTI [5,11,12]. These findings align with current data, where most patients suffering from SUTI were females. Similarly, another study observed that patients aged >50 years were at a higher risk for SUTI, which is almost similar to the mean age of 53 years in our study [13]. Furthermore, we observed that males with SUTI had a longer duration of DM, but the duration of SGLT2I use was similar in both sexes. The median duration of SGLT2I use in this study was 16 weeks in males and 12 weeks in females which is lower than that observed in other studies. In a meta-analysis of 35 randomized controlled trials involving around 35,000 patients, it was found that a dapagliflozin dose of 10 mg/day over 24 weeks increases the risk of UTI [2]. In this study, poor glycemic control, as manifested by HbA1c, considered a risk factor for SUTI, was not different between males and females, but was higher than target levels.

Although patients with DM have an increased risk of UTI, in general [14,15], SGLT2I use worsens this risk further [15-17]. The reason for increased UTI risk in SGLT2I users is attributed to glycosuria, which leads to poor neutrophil mobilization in the urinary tract along with the delayed release of interleukin-6 and interleukin-1b [18]. The majority of SUTIs are mild to moderate and do not warrant discontinuation [8,19], although can have high morbidity at times [20]. In this study, most infections in females were uncomplicated, while among males, around 45% had complicated UTIs such as renal abscesses, pyelonephritis, and prostatic abscesses. Similar reports of adverse outcomes of SUTI in males have been observed previously [10,21,22].

This study provides a fair comparison of the clinical profile of SUTI among males and females. However, this study has some limitations such as a small sample size and the lack of a control group.

## Conclusions

In this cohort of patients with DM, we observed that SUTI was uncomplicated in the majority of females, while in males, one-third had complicated infections. Although females with SUTI had a higher prevalence of obesity and dyslipidemia, males had a longer duration of DM and a higher retinopathy prevalence. Extreme caution should be exercised in patients at risk for SUTI before prescribing SGLT2Is.
